# Implementing advance care planning with community-dwelling frail elders requires a system-wide approach: An integrative review applying a behaviour change model

**DOI:** 10.1177/0269216319845804

**Published:** 2019-05-06

**Authors:** Sarah Combes, Caroline Jane Nicholson, Karen Gillett, Christine Norton

**Affiliations:** 1 Florence Nightingale Faculty of Nursing, Midwifery and Palliative Care, King’s College London, London, UK; 2 St Christopher’s Hospice, London, UK

**Keywords:** Advance care planning, communication, end-of-life care, frail elderly, aged, behavioural change, systematic review

## Abstract

**Background:**

Facilitating advance care planning with community-dwelling frail elders can be challenging. Notably, frail elders’ vulnerability to sudden deterioration leads to uncertainty in recognising the timing and focus of advance care planning conversations.

**Aim:**

To understand how advance care planning can be better implemented for community-dwelling frail elders and to develop a conceptual model to underpin intervention development.

**Design:**

A structured integrative review of relevant literature.

**Data sources:**

CINAHL, Embase, Ovid Medline, PsycINFO, Cochrane Library, and University of York Centre for Reviews and Dissemination. Further strategies included searching for policy and clinical documents, grey literature, and hand-searching reference lists. Literature was searched from 1990 until October 2018.

**Results:**

From 3043 potential papers, 42 were included. Twenty-nine were empirical, six expert commentaries, four service improvements, two guidelines and one theoretical. Analysis revealed nine themes: education and training, personal ability, models, recognising triggers, resources, conversations on death and dying, living day to day, personal beliefs and experience, and relationality.

**Conclusion:**

Implementing advance care planning for frail elders requires a system-wide approach, including providing relevant resources and clarifying responsibilities. Early engagement is key for frail elders, as is a shift from the current advance care planning model focussed on future ceilings of care to one that promotes living well now alongside planning for the future. The proposed conceptual model can be used as a starting point for professionals, organisations and policymakers looking to improve advance care planning for frail elders.

**What is already known about the topic?**

- Frail elders are projected to become one of the largest future users of palliative care.
- Advance care planning can improve person-centred end-of-life care quality; however, it is relatively uncommon in frail elders due to multiple complex challenges.
- Behaviour change models can be used to identify relevant behaviours to inform the development of advance care planning interventions.

**What this paper adds?**

- Implementing advance care planning for frail elders requires a system-wide approach that recognises the importance of living well now, relationality and early engagement.
- All stakeholders (frail elders, families and professionals) have educational needs around the impact of frailty on the life course and why advance care planning is relevant for frail elders.
- The proposed conceptual model can be used as a starting point for professionals, organisations and policymakers looking to improve advance care planning for frail elders.

**Implications for practice, theory or policy**

- Frail elders need to be engaged early with advance care planning to give them the greatest chance to engage physically and cognitively, at their own pace, and make and revise decisions.
- Reframing advance care planning as something that promotes living well now as well as planning for the future would relate more readily to frail elders’ daily lives.
- Professionals need to be given the opportunity to develop the skills and competencies required to recognise, proactively use and create advance care planning facilitation opportunities throughout frail elders’ end-of-life trajectories.

## Background

Frailty is a syndrome of ageing affecting around 10% of those aged above 65,^1^ increasing to around 65% of those aged 90 and above.^2^ Characterised by a progressive, gradual decline in physical, psychological and social functions,^3^ frailty increases vulnerability to sudden deterioration^4,5^ and reduces recovery potential.^6^ Compared to fit older people, those with frailty are at greater risk of disability, care home admission, hospitalisation and death.^7,8^ Frail elders are projected to become one of the largest users of palliative care services,^9^ although currently frail elders are often not recognised as having palliative care needs.^10^

Conversations about end-of-life care, or advance care planning (ACP), are promoted in many high-income countries as a strategy to improve end-of-life care.^11–15^ However, ACP is relatively uncommon in frail elders.^16–18^ Priorities are often not discussed prior to significant deteriorations^19^ when frail elders are unlikely to be able to voice their preferences.^20^ Lack of engagement is due to multiple complex challenges.^16–18^ These include uncertainty of prognostication, therefore recognising when to initiate ACP,^21,22^ misunderstandings around what ACP means,^23^ and frail elders and their families not wanting to discuss death and dying because the topic feels taboo or challenges the frail elders’ coping strategies.^24,25^

One previous review explored ACP in community-dwelling frail elders.^19^ Sharp et al.’s 2013 review,^19^ set within general practice, found that most frail elders would value discussing ACP and that general practitioners recognised ACP as part of their professional responsibility. However, conversations often did not occur due to multiple time pressures and barriers. This integrative review aims to understand how ACP can be better implemented for community-dwelling frail elders (frail elders whose main residence is home or a long-term care facility) and for all relevant multidisciplinary professionals. The review underpins a larger study to develop an intervention to facilitate ACP in this population using the COM-B behaviour change model. The literature analysis is mapped to key stakeholder groups: frail elders; families, including friends and significant others; and professionals, including health and social care professionals.

### Behaviour change theory

**Figure 1.**
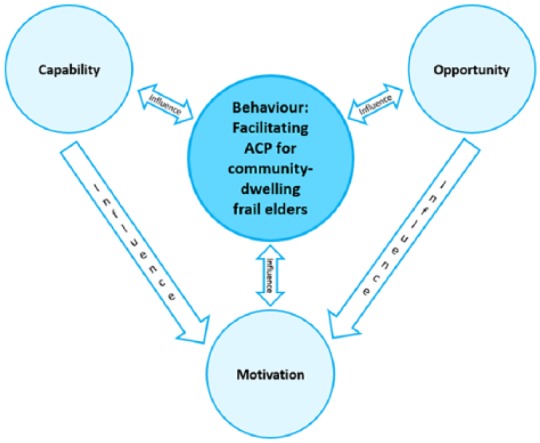
The interlinking elements of behaviour change as proposed by COM-B.^27^

To develop an intervention that successfully influences behaviours to bring about sustained change requires an understanding of current behaviours.^26^ This review uses the COM-B^27^ behaviour change model as a conceptual framework to support the identification of necessary ACP behaviours. COM-B^27^ argues that for a person to change and sustain a change in behaviour, three interlinking elements are required (Figure 1). *Capability* relates to the physical and psychological knowledge and skills required to engage in a behaviour. *Opportunity* relates to physical and social opportunities that exist independently from the individual, such as the environment, resources and interpersonal influences that facilitate a behaviour. *Motivation* relates to an individual’s psychological processes that automatically, or reflectively, direct or encourage the behaviour, including conscious, analytical decision-making and unconscious or habitual responses. COM-B was selected as it is designed to be comprehensive and pragmatic so that it can be used with all behaviours in diverse settings,^28–32^ links to the taxonomy of existing behaviour change theories,^33^ and maps to the larger Behaviour Change Wheel,^34^ thus supporting the translation of behaviour identification into behavioural interventions.^31^

## Method

### Rationale

Whittemore and Knafl’s^35^ systematic integrative review method enabled the synthesis of a wide range of experimental and non-experimental evidence from diverse sources^36–39^ including policy and theoretical documents alongside empirical studies. The narrative synthesis of findings enables a more comprehensive understanding of what is a complex, and at times nebulous, phenomenon.^39^

### Literature search

Multiple search methods were used (Supplementary Data 1). Search terms were developed and refined through a preliminary scoping review and by reviewing key words of relevant papers. The search strategy (Supplementary Data 2) was tailored to each database. Medical Subject Headings (MeSH) were used, where available, to efficiently identify the most relevant data,^40^ alongside free-text synonyms and truncation. The Boolean term ‘OR’ was used to combine multiple terms within a concept and ‘AND’ to combine concepts.^41^ The search, screening and selection, conducted by the first author (S.C.), were verified by the research team, and one author (C.J.N.) completed an independent screen of 10% of papers at both screening and selection stages. Following paper identification and de-duplication, titles and abstracts were screened, and full papers were assessed for eligibility guided by the inclusion criteria (Table 1).

**Table 1.** Inclusion and exclusion criteria.

Inclusion criteria	Exclusion criteria
Adults ⩾65; community-dwelling; living with frailty; cognitively able to discuss ACP; papers that describe the implementation of ACP; 1990 onwards; English language. All data sources.	Acute care settings or papers that only discuss non-acute settings peripherally; papers that only minimally describe the implementation of ACP. Systematic review papers were treated as sources of original papers only.

ACP: advance care planning.

**Figure 2.**
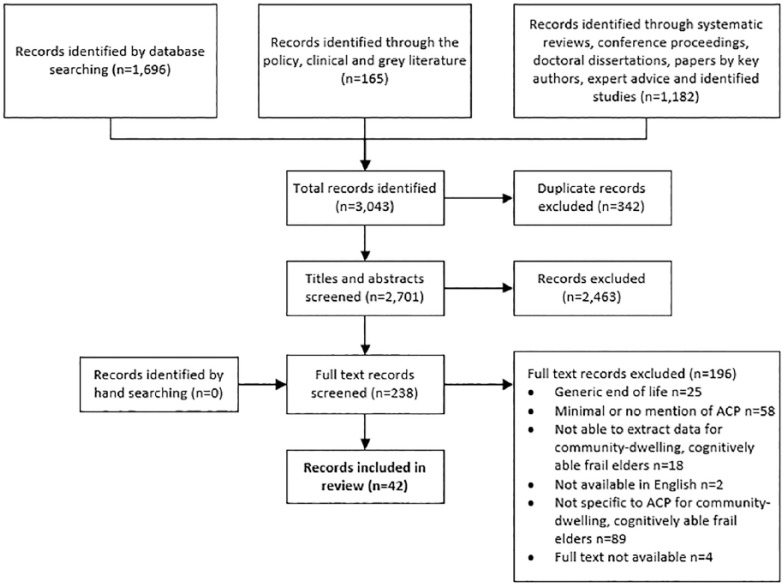
PRISMA: Flow of papers through the selection process.

Searches were limited to papers from 1990 when ACP first appeared in the literature^15^ to October 2018, but not limited by source. The concept of, and process for, quality assessment is complex in integrative reviews with diverse sources, particularly when non-empirical sources are included.^35^ The complexity of this review is increased as multiple conceptualisations of frailty and ACP exist internationally and over time. To ensure all relevant evidence was incorporated, papers were considered based on their relevance to the review’s aim, and so no quality appraisal was conducted. This enabled the inclusion of papers that discussed concepts in their broadest sense, for example, where authors described participants as frail, and residence in long-term care homes was used as a frailty proxy (Figure 2).

### Data analysis

This focussed on the identification and synthesis of attitudes to, and necessary behaviours for, implementing ACP with community-dwelling frail elders. COM-B^27^ was used as a theoretical framework to inform analysis. Using the constant comparison method,^42^ codes and subthemes iteratively emerged within the three COM-B elements of *Capability, Opportunity* and *Motivation.*^27^ Codes were then mapped to the three stakeholder groups (frail elders, families and professionals) to better target behaviours and intervention strategies. Analysis was conducted by S.C. and discussed and reviewed with the research team throughout the process, with final themes agreed by consensus.

## Findings

### Overview

Forty-two papers were included. Ten papers discussed elements of five studies,^43–52^ leaving 37 unique records. Although 22 papers included frail elders as participants, only 10 focused on their views^47,48,53–59^ or those of family members;^59,60^ most focussed on professionals’ experiences and needs. The 29 empirical papers used a range of qualitative, quantitative and mixed methods, including interviews, focus groups, case studies, cohort design, record reviews, quasi-experimental, pilot and randomised controlled trials. Of the 33 empirical and service improvement papers, 11 discussed interventions (Supplementary Table 3). These included reactive case management,^61^ storytelling incorporating reminiscence therapy,^43,44^ a video decision aid,^62^ or versions of previously tested interventions: Let Me Decide,^63^ PEACE (Promoting Effective Advance Care for Elders)^46^ and Respecting Choices.^64–66^ The 42 papers are listed in Table 2.

**Table 2.** Study characteristics.

	*N* = 42 (%)	Reference(s)
Participants (multiple participant types appear in some papers)		
Frail elders	22 (52)	43, 44, 46, 48, 53–56, 58, 59, 61–72
Family	5 (12)	57, 59, 60, 71, 72
Professionals	13 (31)	47, 49–52, 59, 60, 66, 71–75
None (e.g. guidelines, commentary. All focused on professionals)	10 (24)	45, 76–84
Country		
Australia	1 (2)	73
Canada	1 (2)	63
China	2 (5)	43, 44
Italy	1 (2)	59
The Netherlands	1 (2)	66
Norway	2 (5)	53, 71
UK	12 (29)	47, 48, 54, 57, 61, 72, 74, 75, 78, 82–84
USA	22 (53)	45, 46, 49–52, 55, 56, 58, 60, 62, 64, 65, 67–70, 76, 77, 79–81
Year		
1990–1999	5 (12)	55, 63, 68, 69, 80
2000–2005	3 (7)	58, 64, 79
2006–2010	12 (28)	43, 49, 51, 52, 56, 67, 70, 71, 73, 74, 76, 77
2011–2015	15 (36)	44–48, 50, 57, 60–62, 65, 72, 78, 81, 83
2016 onwards	7 (17)	53, 54, 59, 66, 75, 82, 84
Setting		
Home	7 (17)	45, 46, 55, 61, 63, 65, 67
Long-term care	18 (43)	43, 44, 47, 48, 53, 57–60, 62, 69, 70, 72–74, 81, 83, 84
Mixed community	16 (38)	49–52, 56, 64, 66, 68, 71, 75–80, 82
Mixed community and acute	1 (2)	54
Design		
Empirical	29 (69)	
Mixed methods	4 (10)	47, 69, 70, 74
Mixed methods (qualitative reported only)	3 (7)	44, 51, 52
Mixed methods (quantitative reported only)	3 (7)	43, 49, 50
Qualitative	11 (26)	48, 53, 54, 56–60, 71, 72, 75
Quantitative	8 (19)	46, 55, 59, 61–64, 67
Expert commentary	6 (14)	76–81
Guidelines	2 (5)	82, 83
Service improvement	4 (10)	65, 68, 73, 84
Theoretical	1 (2)	45
Interventional study	11 (26)	43, 44, 46, 61–68

The main behavioural components from the narrative synthesis are presented within the three COM-B themes in Table 3 and discussed below. Themes are interrelated, and while they are presented separately here for clarity, they should be considered holistically.

**Table 3.** Attitudes and necessary behaviours for ACP in frail elders.

Theme	Subtheme	Targeted stakeholder	Key messages/influencing factors	References
Capability	Education and training	All	*Strategies and content*: Frail elders/families: ACP relevance; time Professionals: Communication; approaches	44–46, 48–53, 56–58, 62, 64, 65, 67–70, 72, 73, 76–79, 83, 84
	Personal ability	Frail elders and professionals	Frail elders: Early engagement Professionals: Knowledge and skills required to proactively create and use opportunities	43–60, 62–84
Opportunity	Models	All	*Approaches for implementing ACP* ACP as part of everyday practice; integrated, comprehensive, system-wide approach	43–84
	Recognising triggers	Professionals	*Recognising, acting on and creating triggers to engage* Triggers included prognostication; policy/guidelines; environment	43–45, 47–55, 57–67, 69–84
	Resources	Professionals	Engaged leadership; staffing; financial commitment; time; common documentation and retrieval mechanisms; ongoing education and training	43, 45–54, 57, 58, 60–79, 81–84
Motivation	Conversations on death and dying	All	*Barriers to starting/engaging in ACP*: Frail elders: Death as part of life; pace Frail elders/Families: Unrealistic views; language Professionals: Fearing upset/anxiety; taboo	43–45, 48, 51–60, 64–72, 74, 75, 78, 80, 83
	Living day to day	All	Frail elders: Living well now; ambivalence; uncertainty; someone else will decide; autonomy Families: Insecurity	43, 44, 47, 48, 53–57, 59–61, 64, 66–72, 76, 78, 79
	Personal beliefs and experience	All	*Personal values, beliefs, goals and experiences*: All: Previous planning experiences; challenging beliefs Frail elders: Impact of daily life; family burden Professionals: Insecurity; responsibility; feeling undervalued; paternalism	43, 44, 47–52, 54, 56–60, 63–65, 67, 70–76, 78, 79, 84
	Relationality	All	Living within relationships; decision-making in relation; family dynamics; developing trusting relationships.	43–45, 47, 48, 51–60, 64, 65, 67–76, 78–81, 83

ACP: advance care planning.

### Capability: physical and psychological capabilities that enable ACP engagement

Discussed by all but one paper,^61^ this theme was the least nebulous of the three themes and is represented by two subthemes: (1) *Education and training* – packages to improve ACP engagement for all stakeholders, and (2) *Personal ability* – individual knowledge and skills to enable engagement with ACP.

*Education and Training* recommended multiple, diverse formal and informal education and training packages to support stakeholders to better understand, engage with and implement ACP. For frail elders and their families, this included education to improve understanding of ACP,^44,64,69,72^ how to complete documentation,^60^ its relevance for frail elders^53^ and their likely end-of-life trajectory.^44,56,73^ Time was recognised as important for education or training: time to understand necessary concepts and how they related to the frail elder, and time to make decisions.^44,62,64,68,73^ Strategies included making ACP part of routine practice,^57,58,69,73,78^ providing targeted materials,^62,70^ and preparing frail elders and families for potential future decision-making.^48^ For professionals, educational packages focused on improving communication skills and their ability to sensitively engage frail elders with ACP.^45,51,52,57,65,67,69,73,78,79,83,84^ Various educational strategies were recommended, including role-play, online training, role modelling experts, and mentoring.^49,50,72,73,84^ Specific approaches were also discussed, including following a basic palliative approach,^45,76,84^ using core scripts,^77,78^ or attending formal programmes such as Respecting Choices.^46,64^

*Personal ability* related to both frail elders and professionals. For frail elders, it focussed on their physical and psychological ability to engage with ACP and how these would likely reduce with time.^43,45,48–55,57–60,62–64,66,69–72,74–77,79–81,83^ Abilities included difficulties reading and understanding documentation^58,60^ and remembering ACP decisions.^59,60,66,70^ The focus was on early engagement, prior to potential physical or cognitive deterioration^43,45,48,51–55,57–60,62,64,66,71,72,74,75,77,79–81^ where ‘… the person may already be too sick to interpret their treatment preferences’.^43^ Early engagement meant ‘… meaningful plans could be put in place […] so that the patient’s quality of life could be enhanced …’^80^ and that decisions could be reassessed throughout the frail elders’ end-of-life trajectory.^45,48,52,57–59,62–64,69,76,81,83^ For professionals, personal ability related to the knowledge and skills they required to proactively use and create ACP opportunities. Recommendations ranged from needing a greater understanding of what ACP meant,^72^ to the ability to address cultural, socio-demographic and educational influences,^76^ answer existential questions,^65^ help frail elders connect ACP with their own values and beliefs,^56^ and cross-sectoral liaison.^73^

### Capability: key messages

Early engagement means frail elders are most likely to be able to engage with ACP. This is supported by ACP becoming part of everyday practice and the provision of targeted materials. In addition, all stakeholders require access to relevant ongoing education and training. For frail elders, this should focus on understanding what ACP means for them and their likely end-of-life trajectory. For professionals, the focus is on developing the knowledge and skills required to proactively create and use opportunities to engage frail elders in ACP throughout their end-of-life trajectory.

### Opportunity: physical and social opportunities that facilitate ACP

This theme, discussed by all papers, regards the implementation and sustainability of ACP for frail elders. In addition to factors related to the stakeholders, it encompasses organisation and system influences and requirements. The theme represents three subthemes: (1) *Models* – approaches to implementing ACP; (2) *Recognising triggers –* the importance of professionals recognising and utilising ACP triggers; (3) *Resources* – the multiple and diverse resources required for professionals to implement ACP with frail elders.

*Models* related to the various recommended approaches for implementing ACP with frail elders. While it related to all stakeholders, recommendations focussed on professionals, organisations and systems. Several papers focussed on how ACP should be conducted,^45,51,52,58,59,61,64,73,75,77,78,80^ for example, conversations should be ‘… focussed and brief …’,^78^ use open questions ‘what things are most important to you, now and in the future?’,^75^ be held in conducive environments^64^ and include after-death arrangements.^59^ Other papers recommended specific approaches. The recommended palliative, holistic approach^45,46,48,53,54,56,59,60,71,72,76,79^ recognised the importance of relationality (discussed further in *Motivation*), promoted hope, and focussed on living well now rather than planning for dying and death.^53,54,60,72,75^ The storytelling approach included life therapy or using hypothetical scenarios and was promoted as a strategy to support frail elders to clarify their views and beliefs as regards end-of-life wishes.^43,44,48,56,58,62,69^ Integrated and comprehensive system-wide models were seen as important in facilitating ACP.^43,45,53,54,60,64–66,70–75,78^ Recommendations included developing and maintaining cross-sectoral relationships,^47,59,75,84^ ensuring key people, particularly families, were available,^51,52,81^ enabling documentation access,^54,64,65,75^ particularly during care transitions,^60,70,81^ and community-wide support and education.^43,64,66,70,72,74,75^ Almost two-thirds of papers suggested successful ACP necessitated a cross-sectoral, multidisciplinary approach,^45,47,51–54,57,58,60,61,63–65,68–70,73–75,77,80–84^ with the overall recommendation that ACP became ‘… woven into the fabric …’^65^ of everyday practice^43,45,54,58,63,65,73,75,77–79,81^ ‘… as normal as discussing smoking cessation’.^78^

*Recognising triggers* related to professionals recognising, acting on and creating opportunities to engage frail elders.^43,44,47,48,53,54,58–60,62,64,66,67,69–72,75,78,83^ Triggers included recognising poor prognostic indicators,^54,58,61,66,72,77,78,82,83^ transitions, such as admission to homecare services,^45,58,63,67^ and environment, particularly living in long-term care,^43,47,48,53,57–59,72–74,81^ which ‘… allows for continuity of end-of-life care discussion …’^43^ Policy and guidelines that promoted ACP were also triggers,^54,60,67,72–75,78,79,81,83^ particularly when linked to funding or accreditation.^72^ However, there were also multiple barriers. Frailty prognostication is difficult.^47,48,60,75,78,80,83^ The lack of a terminal diagnosis means frail elders ‘… are not identified as being, or do not see themselves as being, at the “end-of-life”’,^48^ especially when they present with ‘… apparent wellness […] during initial consultations …’^47^ Opportunities provided by frail elders were also not always recognised, for example, when a frail elder ‘… refused a percutaneous endoscopic gastrostomy tube and had indicated that he wanted to die …’.^72^ Furthermore, policies and guidelines regarding ACP responsibility were often unclear,^47,51,52,65,71,73,78^ not relevant to frail elders lives,^48,54^ could potentially undermine frail elders’ strategies ‘… to maintain positivity and motivation’,^54^ and often focused on institutional admission with no motivation for ongoing review or relevance to those living in domestic settings.^67^

*Resources* related to the multiple resources required for successful ACP implementation and sustainability, with most discussions including leadership, finance, staffing, time and documentation. Engaged leaders, from commissioners to colleagues, were recognised as important ACP drivers.^48–50,53,63–65,72–74,78,84^ This included supporting professionals to overcome ACP challenges,^49,50,65,72–74,78,84^ enabling resources including funding initiatives and training,^46,47,49–53,57,64,72–74,84^ and employing ‘… a critical mass …’^73^ of trained staff.^43,45,46,54,61,68,71,73,76,78,79,83,84^ Specific professional groups were promoted as ACP facilitators due to their knowledge, skills and responsibilities, for example, nurses,^43,58,63^ general practitioners,^78,81,83^ palliative specialists,^46^ and social workers.^51,52,76^ Other papers suggested successful ACP required round-the-clock community-based exacerbation management teams.^45,61,78^ Time was discussed as a resource by over half the papers.^43,46–53,57,58,63,64,67,69,70,72–75,77,78,81^ For frail elders, this included time to get to know and trust professionals,^48,51,52,57,58,68,69,83^ and ‘… to make the decision, … get information’.^58^ Professionals also required relationship-building time,^51,52,58,76^ and several papers^46,48,51,52,63,70,73^ recommended that organisations allocate staff ‘… the time and skills needed to realistically plan for the future’,^52^ although this was often difficult due to competing priorities.^47,49–52,64,67,72,74,78^ Documentation and the process of completing it was discussed by almost two-thirds of papers.^43,45,47,48,51–54,58,60–65,69,70,72–75,78,79,81–83^ Most recommended ‘… common documents, a common storage and retrieval mechanism …’,^65^ within and across care settings, including frail elders and their families.^45,54,60–62,64,65,69,70,73,75,81–83^ Document contents were also discussed by most authors,^48,51,52,62,63,69,72,74,75,79,81–83^ although there was lack of consensus around whether the document should focus solely on specific treatments^69,79,81^ or recognise personal goals.^43,54,60,72,82^

### Opportunity: key messages

Frail elders are more likely to engage with ACP if it becomes part of everyday practice as part of an integrated, comprehensive, system-wide approach that occurs over time, rather than as a one-off event. Professionals need to recognise, act on, and create opportunities for frail elders to engage with ACP throughout their end-of-life trajectory. To enable this, professionals need support from engaged leaders within their organisations and the wider system, including the provision of all necessary resources such as staffing, finances, education and common documentation.

### Motivation: psychological processes that encourage or direct individual ACP engagement

Discussed by all but four papers,^46,62,77,82^ this theme related to all stakeholders. It is represented by four closely related subthemes: (1) *Conversations on death and dying* – difficulties inherent in engaging with ACP conversations; (2) *Living day to day* – frail elders’ focus on living in the moment rather than planning for the future; (3) *Personal beliefs and experience* – how these influence ACP engagement; (4) *Relationality* – the impact of living within relationships.

The subtheme *Conversations on death and dying*, discussed by almost two-thirds of all papers, raised important barriers around starting conversations and engaging in informed decision-making.^43–45,48,51–60,64–72,74,75,78,80,83^ Frail elders and families held wide-ranging ACP views from rejection to full engagement,^44,48,59,66^ with many frail elders viewing death as part of life.^43,44,53,55,69,72^ While ACP had the potential to cause distress or make frail elders initially ‘… slightly uncomfortable …’,^53^ most saw ACP as ‘… a welcome intervention …’,^59^ as long as conversations were at the frail elder’s pace.^48,59,66,68,83^ For professionals, barriers included struggles discussing a taboo subject^44,51–53,57,71,72^ and fear of causing suffering or anxiety.^43,53,55,57,67,69,71,72,75,80^ Informed decision-making could also be challenging. Language could be confusing for frail elders and families, notably legal requirements, documentation^54,58,60,64^ and language around ACP, particularly what it meant,^45,58,59,67,69,78^ with many fearing ACP was ‘… irrevocable…’ and led to professional ‘… abandonment …’^69^ Informed decision-making was also impacted by unrealistic views, including misunderstanding what medical treatments or palliative care would likely achieve,^48,55,67,71^ the availability of services and support,^48,54,75^ the frail elder’s ability to recover, and denial that the frail elder was nearing the end of life.^51,57,59,71^ Families found this particularly difficult when they felt they had been given ‘… irrational optimism …’ regarding prognosis.^60^

*Living day to day*, discussed by over half the papers, related to how frail elders focussed on living well now, maintaining quality of life, rather than on future planning.^43,44,47,48,53–57,59–61,64,66–72,76,78,79^ While some frail elders appreciated ACP as a way to ‘… express their opinion …’,^59^ there was an ambivalence around ACP. Frail elders often did not see how ACP could be relevant when likely rapid physical or psychological deterioration ‘… meant that any plans may become obsolete quite quickly’.^54^ Frail elders often trusted family or professionals to make future care decisions in their best interests,^43,44,48,53,54,56,59,68,69,72,78^ as these ‘others’ knew what they wanted, challenging the concept of autonomy as a motivating factor for ACP engagement. In reality, while some families felt they knew the person’s preferences,^71^ most felt insecure making decisions as preferences had not been discussed:^44,48,53,54,60,68,69,72,78^ ‘It’s hard to be the healthcare proxy […] you say, “Am I doing the right thing?”’^60^

*Personal beliefs and experiences* discussed how ACP motivation largely related to personal beliefs, values, goals, and experiences and how these, and therefore motivation to engage with ACP, can change over time. For all stakeholders, previous future planning experiences, such as helping others make end-of-life decisions,^56^ facilitating ACP,^49,50,58,75^ or having experience with the dying process,^60,72^ could encourage or discourage engagement. For frail elders, personal beliefs included whether they believed decisions would impact their day-to-day life,^48,59^ distrusting the proxy process,^59^ or a desire not to burden their family.^56^ For professionals, papers mainly discussed demotivating beliefs, including that ACP conversations were ‘… undervalued …’^72^ by colleagues or managers, that professionals lacked the confidence to manage complex, often upsetting conversations,^51,52^ and concern that ‘… lack of services’ would impact ACP implementation.^75^ Many professionals expressed paternalism, wishing to make decisions themselves as they feared ACP conversations would upset frail elders,^43,67^ burden families,^71^ or challenge their sense of patient responsibility.^54,64,78^ Responsibility for ACP was unclear,^47,51,52,57,59,73,74^ with many professionals reluctant to assume responsibility,^51,52,57^ believing ACP was within another professional’s remit.^57^ This highlighted the need to be ‘… more discriminating about who is responsible for which elements of ACP practice …’^74^

*Relationality*, discussed by almost three-quarters of papers, related to how frail elders live within relationships, whether family, friends, professionals or cultures,^43–45,47,48,51–60,65,67–69,71–76,78–81,83^ and the impact relationality had on ACP decision-making. Relationality included frail elders wanting to make decisions within relationships^43,48,54,56–59,75^ and being more concerned with how ACP decisions may affect others than themselves.^48,56,58^ Developing trusting relationships, particularly the frail elder/professional relationship,^43,44,47,48,51–58,60,64,68–72,76,78,80,81,83^ was recognised as important, with the development of rapport and trust between all stakeholders cited by many as ‘… the cornerstone …’^44^ of ACP engagement. Long-term care homes were considered excellent environments for this. However, opportunities for professionals to build trusting relationships with frail elders living at home were less promising due to the ‘… erosion of personal continuity between a doctor and their patient …’.^75^ Other challenges included disagreements within families,^65,67,71^ between the frail elder and their family^43,47,51,52,58,65,71,74,80^ or between families and professionals.^57^ Further difficulties were caused by lack of or limited family involvement^45,47,48,51,52,59,60,72,74,76,79,81^ and limited social networks.^51,52,79^

### Motivation: key messages

The importance of relationality and living well now should be recognised by all stakeholders, with frail elders supported to make decisions within relationships should they wish. Professionals should attempt to build trusting relationships with frail elders and their families as appropriate. In addition, professionals should assess frail elders’ readiness to engage, clarify misunderstandings, and work with them at their own pace. To enable this, professionals need greater clarity around ACP responsibilities and require support to challenge any stakeholder’s negative personal beliefs.

## Discussion

### Main findings

**Figure 3.**
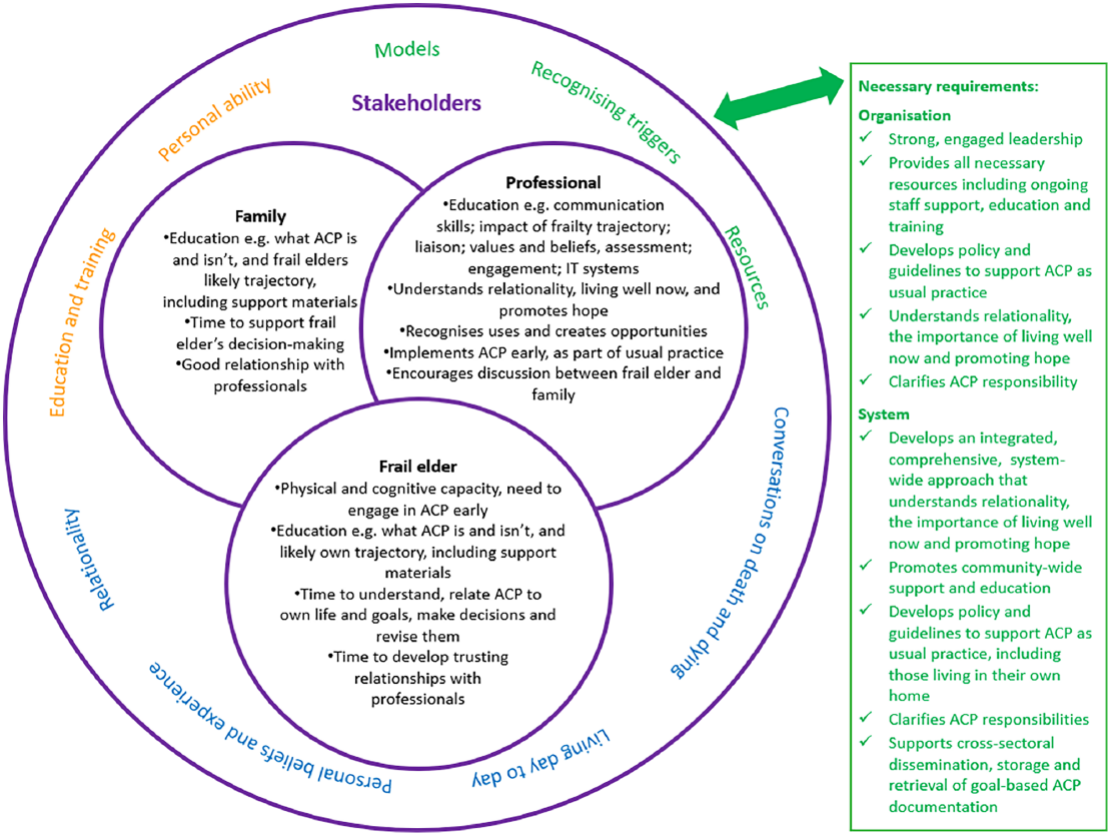
Conceptual model of the behaviours, factors and requirements necessary to conduct successful ACP with community-dwelling frail elders.

The main behavioural components, factors and requirements necessary to conduct successful ACP with community-dwelling frail elders are represented conceptually below (Figure 3). This proposed conceptual model includes the interrelated stakeholder behaviours and factors as well as the organisational and system requirements found to be important influencers particularly in enabling professional’s opportunity behaviours.

The model demonstrates the complexity inherent in ACP facilitation for community-dwelling frail elders, with all elements required for successful ACP implementation. It recommends an approach that recognises the importance of early engagement, relationality, living well now, and stakeholder education, particularly educating professionals to develop the knowledge and skills required to recognise, create and use ACP facilitation triggers throughout frail elders’ end-of-life trajectories. These core recommendations are discussed further below.

### Capability

Early engagement, which provides frail elders the greatest chance of being able to engage physically and cognitively with ACP, is the key Capability. If successful, early engagement enables frail elders to engage at their own pace, to understand necessary concepts and how these relate to them, to put meaningful plans in place, to revise decision-making, and to develop trusting relationships with professionals. While finding the ‘right time’ to start ACP is difficult,^6,21^ early engagement promotes the concept of ACP as an ongoing process that takes place over time and is revisited regularly throughout the frail elders’ life course.^85,86^ To enable early engagement, all stakeholders require ongoing education. Notably, professionals needed to develop the knowledge and skills required to recognise, create and use triggers to facilitate ACP throughout frail elders’ end-of-life trajectories. Evidence suggests that professionals often require knowledge and communication-based training to enable ACP^85^ as conversations can be challenging.^87^ The requirement to create and use triggers is also strongly influenced by Opportunity, as frail elders often have little professional contact and few perceived end-of-life needs.^16,88^

### Opportunity

ACP as part of everyday practice and something that occurs over time, rather than a single event, is the key Opportunity. This is reflected in the model’s recommendation of the vital necessity of a system-wide approach, and with it, the necessary resources to support it at every level. This takes forward Sharp et al.’s^19^ review which suggested policymakers and healthcare professionals need to address multiple issues to promote personal autonomy, such as informed decision-making, within healthcare systems with limited resources. The concept of an integrative, comprehensive, system-wide approach to ACP for frail elders supports the call for more integrated care systems to better meet the needs of older people.^89^ The model also aligns with the public health palliative care approach, which raises community awareness and engagement with end-of-life issues and influences social views of death and dying.^90^ This approach presents an opportunity for behavioural change in ACP and may help challenge the conceptualisation of ACP as a failure of medical care by some.^91^ As with this public health palliative movement, to effect long-term ACP change requires a system-wide approach, incorporating national campaigns and policy, through to the involvement and commitment of multiple community leaders, organisations and individuals.^92^

### Motivation

Relationality and living well now are the key Motivations. Individual autonomy is promoted throughout current ACP policies and literature and the current measurable activities system of incentivising health and social care.^91^ However, the model challenges the concept of autonomy as the sole motivating factor for frail elders’ ACP engagement. The findings demonstrate the importance frail elders place on living and making decisions within relationships, sometimes choosing that others will make ACP decisions for them. This review demonstrates that often frail elders focus on maintaining current quality of life rather than on ACP, with future planning seen as irrelevant for some within the context of their uncertain physical and psychological trajectory. This links strongly with Capability and Opportunity, emphasising the need to start conversations early and continue them over time, providing frail elders opportunities to change their views as their trajectory changes. These findings are supported by much of the ageing literature which suggests that many older people prioritise trusting relationships and relational decision-making over autonomy,^85,93,94^ valuing living well now above future planning.^23,95^ Reframing ACP for frail elders to become something that promotes living well now in addition to future planning and recognising the importance of relational autonomy by supporting frail elders to make decisions within relationships would relate more readily to their daily lives. This reframing may be key to successful ACP implementation in this population.

### Behavioural change theory

The model calls for system-wide, multi-level implementation, the recommended approach for successful behaviour change interventions,^96^ but current health and social care resource limitations mean this is challenging. While policy, for example, the UK long-term care plan, demonstrates the importance of personalised care at end of life, the UK community sector is experiencing increasing workloads, patient complexity, and lack of funding.^97,98^ This has led to the prioritisation of core care, such as diagnosis and treatment, over more holistic needs^99^ such as ACP. The global picture is similar, particularly regarding end-of-life and palliative care,^100^ with priorities often focussed on more fundamental needs such as access to analgesia.^101^ However, an incremental approach, making pragmatic decisions by focussing on fewer behaviour changes and building on the success of these,^34^ can also be used to facilitate ACP with frail elders. This strategy can be demonstrated by the concept of early engagement, which could be supported by making ACP part of everyday practice and providing targeted materials for frail elders to read in their own time. This would reduce the need for professional involvement at every step of the decision-making process, thus minimising the use of health and social care resources, and as a by-product could promote trusting relationships and relational decision-making.

### Strengths and limitations

The proposed conceptual model is limited by the literature. The study exclusion criteria may have meant some relevant papers were missed, particularly papers prior to 1990, those not in English, and those where older participants self-identified as healthy. The use of a proxy for frailty, particularly the proxy of residence in long-term care homes, may have skewed the data away from the needs of frail elders living in domestic settings. The voices of frail elders and families were reduced as literature mainly focussed on behaviours and factors influencing professionals. Furthermore, minimal literature explored early engagement, public health models or moving beyond professional responsibility for ACP within this population. The strength of the review, and therefore the proposed conceptual model, is its rigorous methods; use of a research team to discuss, review and verify the process; and the use of a theoretical model. Using COM-B^27^ as a framework ensured a focus on implementation throughout the review and synthesis, and that individual- and system-level behaviours were considered. While some behaviours were influenced by more than one COM-B element, this demonstrates the complexity of the topic and the multidimensional, interdependent behaviours that require targeting for the success of any whole-system intervention.

### Implications for clinical practice and research

The conceptual model can be used as a starting point for professionals, organisations and policymakers when looking to improve ACP for frail elders. The themes and key necessary requirements are displayed at stakeholder, organisation and system levels to help target relevant behaviours or requirements depending on the reader’s purpose. This is demonstrated above with the example of early engagement. Further targets that do not require significant health and social care resource but are likely to have a significant impact on successful ACP facilitation are providing opportunities for professionals to develop skills to recognise, proactively use and create facilitation opportunities; professionals’ understanding and working with relationality, including developing trusting relationships and enabling relational decision-making; and reframing ACP for frail elders to focus on living well now as well as future planning.

## Conclusion

This review is the first to define the necessary requirements to enable ACP for community-dwelling frail elders and synthesise these into a proposed conceptual model. The model can be used as a starting point for professionals, organisations and policymakers looking to improve ACP for community-dwelling frail elders. Key messages are that frail elders should be engaged early in the process of ACP, that ACP should be reframed as a discussion of current care goals as well as future planning, and that professionals need the opportunity to develop the skills and competencies required to recognise, proactively use and create ACP opportunities throughout frail elders’ end-of-life trajectories. Further research will focus on refining and testing the model in practice, prior to collaborative intervention development with stakeholders.

## Supplementary Material

Supplementary material

Supplementary material

Supplementary material

## References

[1] Clegg A Young J Iliffe S et al Frailty in elderly people. Lancet 2013; 381: 752–762. 23395245 10.1016/S0140-6736(12)62167-9 PMC4098658

[2] Gale CR Cooper C Sayer AA. Prevalence of frailty and disability: findings from the English longitudinal study of ageing. Age Ageing 2015; 44(1): 162–165. 25313241 10.1093/ageing/afu148 PMC4311180

[3] Van Campen C Frail older persons in the Netherlands. The Hague: The Netherlands Institute for Social Research, 2011.

[4] Covinsky KE Eng C Lui L et al The last 2 years of life: functional trajectories of frail older people. J Am Geriatr Soc 2003; 51(4): 492–498. 12657068 10.1046/j.1532-5415.2003.51157.x

[5] Turner G Clegg A, British Geriatrics Society, et al Best practice guidelines for the management of frailty: a British Geriatrics Society, Age UK and Royal College of General Practitioners report. Age Ageing 2014; 43(6): 744–747. 25336440 10.1093/ageing/afu138

[6] Nicholson C Morrow EM Hicks A et al Supportive care for older people with frailty in hospital: an integrative review. Int J Nurs Stud 2016; 66: 60–71. 28012311 10.1016/j.ijnurstu.2016.11.015

[7] Fried LP Tangen CM Walston J et al Frailty in older adults: evidence for a phenotype. J Gerontol A Biol Sci Med Sci 2001; 56(3): M146–M156. 11253156 10.1093/gerona/56.3.m146

[8] Rockwood K Mitnitski A Song X et al Long-term risks of death and institutionalization of elderly people in relation to deficit accumulation at age 70. J Am Geriatr Soc 2006; 54(6): 975–979. 16776795 10.1111/j.1532-5415.2006.00738.x

[9] Bone AE Gomes B Etkind SN et al What is the impact of population ageing on the future provision of end-of-life care? Population-based projections of place of death. Palliat Med 2018; 32(2): 329–336. 29017018 10.1177/0269216317734435 PMC5788077

[10] Lloyd A Kendall M Carduff E et al Why do older people get less palliative care than younger people. ? European J Palliat Care 2016; 23: 132–137.

[11] Department of Health. Our commitment to you for end of life care. The government response to the review of choice in end of life care, www.gov.uk/government/uploads/system/uploads/attachment_data/file/536326/choice-response.pdf (2016, accessed 26 October 2018).

[12] Department of Health. The end of life care strategy, promoting high quality care for all adults at the end of life. London: Department of Health, 2008.

[13] National Palliative and End of Life Care Partnership. Ambitions for palliative and end of life care: a national framework for local action 2015-2020, www.endoflifecareambitions.org.uk/wp-content/uploads/2015/09/Ambitions-for-Palliative-and-End-of-Life-Care.pdf (2014, accessed 28 October 2018).

[14] Henry C. What’s important to me: a review of choice in end of life care, www.gov.uk/government/uploads/system/uploads/attachment_data/file/407244/CHOICE_REVIEW_FINAL_for_web.pdf (2015, accessed 28 October 2018).

[15] Thomas K Lobo B. Advance care planning in end of life care. Oxford: Oxford University Press, 2011.

[16] Musa I Seymour J Narayanasamy MJ et al A survey of older peoples’ attitudes towards advance care planning. Age Ageing 2015; 44(3): 371–376. 25917242 10.1093/ageing/afv041

[17] Pollock K Wilson E. Care and communication between health professionals and patients affected by severe or chronic illness in community care settings: a qualitative study of care at the end of life. Report no. 3, 7 2015 Southampton: Health Services and Delivery Research. 26247082

[18] Brinkman-Stoppelenburg A Rietjens JA van der Heide A. The effects of advance care planning on end-of-life care: a systematic review. Palliat Med 2014; 28(8): 1000–1025. 24651708 10.1177/0269216314526272

[19] Sharp T Moran E Kuhn I et al Do the elderly have a voice? Advance care planning discussions with frail and older individuals: a systematic literature review and narrative synthesis. Br J Gen Pract 2013; 63(615): e657–e668. 24152480 10.3399/bjgp13X673667 PMC3782798

[20] Morley JE Vellas B van Kan GA et al Frailty consensus: a call to action. J Am Med Dir Assoc 2013; 14: 392–397. 23764209 10.1016/j.jamda.2013.03.022 PMC4084863

[21] Clarke A Seymour J. ‘At the foot of a very long ladder’: discussing the end of life with older people and informal caregivers. J Pain Symptom Manage 2010; 40(6): 857–869. 20813494 10.1016/j.jpainsymman.2010.02.027

[22] Pollock K. Is home always the best and preferred place of death. BMJ 2015; 2015: 351. 10.1136/bmj.h4855 26446163

[23] Seymour J Gott M Bellamy G et al Planning for the end of life: the views of older people about advance care statements. Soc Sci Med 2004; 59(1): 57–68. 15087143 10.1016/j.socscimed.2003.10.005

[24] Nicholson C Meyer J Flatley M et al Living on the margin: understanding the experience of living and dying with frailty in old age. Soc Sci Med 2012; 75(8): 1426–1432. 22800918 10.1016/j.socscimed.2012.06.011

[25] Lloyd A Kendall M Starr JM et al Physical, social, psychological and existential trajectories of loss and adaptation towards the end of life for older people living with frailty: a serial interview study. BMC Geriatr 2016; 16(1): 176. 27765011 10.1186/s12877-016-0350-y PMC5072327

[26] Scherrens A Beernaert K Robijn L et al The use of behavioural theories in end-of-life care research: a systematic review. Palliat Med 2018; 32(6): 1055–1077. 29569998 10.1177/0269216318758212

[27] Michie S van Stralen MM West R. The behaviour change wheel: a new method for characterising and designing behaviour change interventions. Implement Sci 2011; 6: 42. 21513547 10.1186/1748-5908-6-42 PMC3096582

[28] Thompson LM Diaz-Artiga A Weinstein JR et al Designing a behavioral intervention using the COM-B model and the theoretical domains framework to promote gas stove use in rural Guatemala: a formative research study. BMC Pub Heal 2018; 18(1): 253. 10.1186/s12889-018-5138-x PMC5813324 29444650

[29] Alexander KE Brijnath B Mazza D. Barriers and enablers to delivery of the Healthy Kids Check: an analysis informed by the Theoretical Domains Framework and COM-B model. Implement Sci 2014; 9: 60. 24886520 10.1186/1748-5908-9-60 PMC4047437

[30] Barker F Atkins L de Lusignan S. Applying the COM-B behaviour model and behaviour change wheel to develop an intervention to improve hearing-aid use in adult auditory rehabilitation. Int J Audiol 2016; 55(Suppl. 3): S90–S98. 27420547 10.3109/14992027.2015.1120894

[31] Jackson C Eliasson L Barber N et al Applying COM-B to medication adherence: a suggested framework for research and interventions. Euro Heal Psychol 2014; 16: 7–17.

[32] Walsh DM Moran K Cornelissen V et al The development and codesign of the PATHway intervention: a theory-driven eHealth platform for the self-management of cardiovascular disease. Transl Behav Med 2019; 9: 76–98. 29554380 10.1093/tbm/iby017

[33] Michie S Richardson M Johnston M et al The behavior change technique taxonomy (v1) of 93 hierarchically clustered techniques: building an international consensus for the reporting of behavior change interventions. Ann Behav Med 2013; 46(1): 81–95. 23512568 10.1007/s12160-013-9486-6

[34] Michie S Atkins L West R. The behaviour change wheel: a guide to designing interventions. Surrey: Silverback Publishing, 2014.

[35] Whittemore R Knafl K. The integrative review: updated methodology. J Adv Nurs 2005; 52(5): 546–553. 16268861 10.1111/j.1365-2648.2005.03621.x

[36] Souza MT Silva MD Carvalho R. Integrative review: what is it? How to do it? Einstein (São Paulo) 2010; 8: 102–106. 26761761 10.1590/S1679-45082010RW1134

[37] Soares CB Hoga LAK Peduzzi M et al Integrative review: concepts and methods used in nursing. Rev Esc Enferm USP 2014; 48(2): 335–345. 24918895 10.1590/s0080-6234201400002000020

[38] Cooper H. The integrative research review: a systematic approach (Applied Social Research Methods Series, vol. 2). Beverly Hills, CA: SAGE, 1984.

[39] Broome ME. Integrative literature reviews for the development of concepts. In: Rogers BL Knafl KA (eds) Concept development in nursing: foundations, techniques and applications. Philadelphia, PA: WB Saunders Company, 2000, pp. 193–216.

[40] Lee DH Schleyer T. Social tagging is no substitute for controlled indexing: a comparison of medical subject headings and CiteULike tags assigned to 231,388 papers. J Assoc Inform Sci Technol 2012; 63: 1747–1757.

[41] Ely C Scott I. Essential study skills for nursing. Edinburgh: Elsevier, 2007.

[42] Miles MB Huberman AM Saldana J. Qualitative data analysis: a methods source book. 3rd ed. Thousand Oaks, CA: SAGE, 2013.

[43] Chan HYL Pang SM Let me talk – an advance care planning programme for frail nursing home residents. J Clin Nurs 2010; 19: 3073–3084. 21040013 10.1111/j.1365-2702.2010.03353.x

[44] Chan HYL Pang SM Readiness of Chinese frail old age home residents towards end-of-life care decision making. J Clin Nurs 2011; 20: 1454–1461. 21492285 10.1111/j.1365-2702.2010.03670.x

[45] Allen KR Hazelett SE Radwany S et al The promoting effective advance care for elders (PEACE) randomized pilot study: theoretical framework and study design. Popul Health Manag 2012; 15: 71–77. 22088165 10.1089/pop.2011.0004

[46] Radwany SM Hazelett SE Allen KR et al Results of the promoting effective advance care planning for elders (PEACE) randomized pilot study. Popul Health Manag 2014; 17: 106–111. 24156664 10.1089/pop.2013.0017

[47] Handley M Goodman C Froggatt K et al Living and dying: responsibility for end-of-life care in care homes without on-site nursing provision-a prospective study. Health Soc Care Community 2014; 22(1): 22–29. 23718786 10.1111/hsc.12055

[48] Mathie E Goodman C Crang C et al An uncertain future: the unchanging views of care home residents about living and dying. Palliat Med 2012; 26(5): 734–743. 21697261 10.1177/0269216311412233

[49] Black K. Correlates of case managers’ advance care planning practices. Clin Gerontol 2010; 33: 124–135.

[50] Black K. Professional and personal factors associated with gerontological practice: implications for training and education. Edu Gerontol 2011; 37: 982–994.

[51] Black K. Exploring case managers’ advance care planning practices. J Soc Serv Res 2007; 33: 21–30. 10.1300/J027v26n02_03 17537710

[52] Black K Fauske J. Exploring influences on community-based case managers’ advance care planning practices: facilitators or barriers? Home Health Care Serv Q 2007; 26: 41–58. 17537710 10.1300/J027v26n02_03

[53] Bollig G Gjengedal E Rosland JH. They know! Do they? A qualitative study of residents and relatives views on advance care planning, end-of-life care, and decision-making in nursing homes. Palliat Med 2016; 30(5): 456–470. 26396227 10.1177/0269216315605753 PMC4838176

[54] Bramley L. One day at a time: living with frailty: implications for the practice of advance care planning: a multiple case study. PhD Thesis, University of Nottingham, Nottingham, 2016.

[55] Kellogg FR Crain M Corwin J et al Life-sustaining interventions in frail elderly persons: talking about choices. Arch Intern Med 1992; 152(11): 2317–2320. 1444692

[56] Levi BH Dellasega C Whitehead M et al What influences individuals to engage in advance care planning? Am J Hosp Palliat Care 2010; 27(5): 306–312. 20103783 10.1177/1049909109355280 PMC3766750

[57] Stewart F Goddard C Schiff R et al Advanced care planning in care homes for older people: a qualitative study of the views of care staff and families. Age Ageing 2011; 40(3): 330–335. 21345840 10.1093/ageing/afr006

[58] White C. An exploration of decision-making factors regarding advance directives in a long-term care facility. J Am Acad Nurse Pract 2005; 17(1): 14–20. 15679879 10.1111/j.1041-2972.2005.00005.x

[59] Ingravallo F Mignani V Mariani E et al Discussing advance care planning: insights from older people living in nursing homes and from family members. Int Psychogeriatr 2018; 30(4): 569–579. 28988561 10.1017/S1041610217001983

[60] Jackson J White P Fiorini J et al Family perspectives on end-of-life care: a metasynthesis. J Hosp Palliat Nurs 2012; 14: 303–311.

[61] Baker A Leak P Ritchie LD et al Anticipatory care planning and integration: a primary care pilot study aimed at reducing unplanned hospitalisation. Br J Gen Pract 2012; 62(595): e113–e120. 22520788 10.3399/bjgp12X625175 PMC3268490

[62] Volandes AE Brandeis GH Davis AD et al A randomized controlled trial of a goals-of-care video for elderly patients admitted to skilled nursing facilities. J Palliat Med 2012; 15(7): 805–811. 22559905 10.1089/jpm.2011.0505 PMC3387760

[63] Patterson C Molloy DW Guyatt GH et al Systematic implementation of an advance health care directive in the community. Can J Nurs Adm 1997; 10(2): 96–108. 9384018

[64] Schwartz CE Wheeler HB Hammes B et al Early intervention in planning end-of-life care with ambulatory geriatric patients: results of a pilot trial. Arch Intern Med 2002; 162(14): 1611–1618. 12123405 10.1001/archinte.162.14.1611

[65] Boettcher I Turner R Briggs L. Telephonic advance care planning facilitated by health plan case managers. Palliat Support Care 2015; 13(3): 795–800. 24914566 10.1017/S1478951514000698

[66] Overbeek A Korfage IJ Jabbarian LJ et al Advance care planning in frail older adults: a cluster randomized controlled trial. J Am Geriatr Soc 2018; 66(6): 1089–1095. 29608789 10.1111/jgs.15333

[67] Golden AG Corvea MH Dang S et al Assessing advance directives in the homebound elderly. Am J Hosp Palliat Care 2009; 26(1): 13–17. 18843136 10.1177/1049909108324359

[68] Luptak MK Boult C. A method for increasing elders’ use of advance directives. Gerontologist 1994; 34(3): 409–412. 8076885 10.1093/geront/34.3.409

[69] Palker NB Nettles-Carson B. The prevalence of advance directives: lessons from a nursing home. Nurse Pract 1995; 20(2): 7–21. 7715868

[70] Yung VY Walling AM Min L et al Documentation of advance care planning for community-dwelling elders. J Palliat Med 2010; 13: 861–867. 20618087 10.1089/jpm.2009.0341 PMC2939845

[71] Schaffer MA. Ethical problems in end-of-life decisions for elderly Norwegians. Nurs Ethics 2007; 14(2): 242–257. 17425152 10.1177/0969733007073707

[72] Stone L Kinley J Hockley J. Advance care planning in care homes: the experience of staff, residents, and family members. Int J Palliat Nurs 2013; 19(11): 550–557. 24263899 10.12968/ijpn.2013.19.11.550

[73] Blackford J Strickland E Morris B. Advance care planning in residential aged care facilities. Contemp Nurse 2007; 27: 141–151. 18386964 10.5555/conu.2007.27.1.141

[74] Froggatt K Vaughan S Bernard C et al Advance care planning in care homes for older people: an English perspective. Palliat Med 2009; 23(4): 332–338. 19324923 10.1177/0269216309103802

[75] Sharp T Malyon A Barclay S. GPs’ perceptions of advance care planning with frail and older people: a qualitative study. Br J Gen Pract 2018; 68(666): e44–e53. 29255110 10.3399/bjgp17X694145 PMC5737319

[76] Black K. Advance care planning throughout the end-of-life: focusing the lens for social work practice. J Soc Work End Life Palliat Care 2007; 3(2): 39–58. 10.1300/J457v03n02_04 18069622

[77] Boockvar KS Meier DE. Palliative care for frail older adults: ‘there are things I can’t do anymore that I wish I could. . . ’. JAMA 2006; 296: 2245–2253. 17090771 10.1001/jama.296.18.2245

[78] Eynon T Lakhani MK Baker R. Never the right time: advance care planning with frail and older people. Br J Gen Pract 2013; 63(615): 511–512. 24152450 10.3399/bjgp13X673568 PMC3782768

[79] Norlander L. The future of advance care planning. Home Health Care Manage Pract 2003; 15: 136–139.

[80] Zuckerman C. Issues concerning end-of-life care. J Long Term Home Health Care 1997; 16: 26–34. 10168380

[81] Zweig SC Popejoy LL Parker-Oliver D et al The physician’s role in patients’ nursing home care: ‘She’s a very courageous and lovely woman. I enjoy caring for her’. JAMA 2011; 306(13): 1468–1478. 21972309 10.1001/jama.2011.1420

[82] British Geriatric Society. Fit for frailty part 1: consensus best practice guidance for the care of older people living in community and outpatient settings, https://www.bgs.org.uk/sites/default/files/content/resources/files/2018-05-23/fff_full.pdf (2014, accessed 28 October 2018).

[83] Guidelines and Audit Implementation Network. Guidelines for palliative and end of life care in nursing homes and residential care homes, www.hscbereavementnetwork.hscni.net/wp-content/uploads/2015/05/GAIN-Guidelines-for-Palliative-and-End-of-Life-Care-in-Nursing-Homes-Residential-Care-Homes-Appendix-2-Revised-September-2014.pdf (2014, accessed 28 October 2018).

[84] Kinley J Stone L Butt A et al Developing, implementing and sustaining an end-of-life care programme in residential care homes. Int J Palliat Nurs 2017; 23(4): 186–193. 28486068 10.12968/ijpn.2017.23.4.186

[85] Godfrey M Hackett J. Advanced care planning: policy and real-life decision-making. Age Ageing 2015; 44(3): 348–350. 25917241 10.1093/ageing/afv046

[86] Taneja R Faden LY Schulz V et al Advance care planning in community dwellers: a constructivist grounded theory study of values, preferences and conflicts. Palliat Med 2018; 33: 66–73. 30284950 10.1177/0269216318803487

[87] Parry R Land V Seymour J. How to communicate with patients about future illness progression and end of life: a systematic review. BMJ Support Palliat Care 2014; 4(4): 331–341. 10.1136/bmjspcare-2014-000649 PMC4251180 25344494

[88] Care Quality Commission. A different ending: addressing inequalities in end of life care, https://www.cqc.org.uk/sites/default/files/20160505%20CQC_EOLC_GoodPractice_2016_FINAL.pdf (2016, accessed 28 October 2018).

[89] Oliver D Foot C Humphries R. Making our health and care systems fit for an ageing population. London: King’s Fund, 2014. 10.1093/ageing/afu105 25074536

[90] Sallnow L Richardson H Murray SA et al The impact of a new public health approach to end-of-life care: a systematic review. Palliat Med 2016; 30(3): 200–211. 26269324 10.1177/0269216315599869

[91] Borgstrom E. Advance care planning: between tools and relational end-of-life care. BMJ Support Palliat Care 2015; 5(3): 216–217. 10.1136/bmjspcare-2015-000979 26290096

[92] Matthiesen M Froggatt K Owen E et al End-of-life conversations and care: an asset-based model for community engagement. BMJ Support Palliat Care 2014; 4(3): 306–312. 10.1136/bmjspcare-2013-000516 24644202

[93] Nolan MR Davies S Brown J et al Beyond ‘person-centred’ care: a new vision for gerontological nursing. J Clin Nurs 2004; 13(3a): 45–53. 15028039 10.1111/j.1365-2702.2004.00926.x

[94] Seymour J. Looking back, looking forward: the evolution of palliative and end-of-life care in England. Mortality 2012; 17: 1–17.

[95] Detering KM Hancock AD Reade MC et al The impact of advance care planning on end of life care in elderly patients: randomised controlled trial. BMJ 2010; 340: c1345. 20332506 10.1136/bmj.c1345 PMC2844949

[96] National Institute for Health and Care Excellence. Behaviour change at population, community and individual levels. NICE Public Health Guidance 6, https://www.nice.org.uk/guidance/Ph6 (2007, accessed 28 October 2018).

[97] Baird B Charles A Honeyman M et al Understanding pressures in general practice. London: King’s Fund, 2016.

[98] Maybin J Charles A Honeyman M. Understanding quality in district nursing services. London: King’s Fund, 2016.

[99] Robertson R Wenzel L Thompson J et al Understanding NHS financial pressures. How are they affecting patient care? London: King’s Fund, 2016.

[100] Worldwide Palliative Care Alliance. Global atlas of palliative care at the end of life. Geneva: World Health Organization, 2014.

[101] Harding R Simms V Penfold S et al Availability of essential drugs for managing HIV-related pain and symptoms within 120 PEPFAR-funded health facilities in East Africa: a cross-sectional survey with onsite verification. Palliat Med 2014; 28(4): 293–301. 23885009 10.1177/0269216313498637

