# Supplementary material for: Implementing advance care planning with community-dwelling frail elders requires a system-wide approach: An integrative review applying a behaviour change model
Source: Palliat Med. 2019 May 6;33(7):743–56. doi: 10.1177/0269216319845804 (PMC6620766; doi:10.1177/0269216319845804)
Supplement: Supplementary material [file Supplementary_Data_1_-_22.03.19.docx]

**Supplementary Data 1: Search Methods**

- **Database searches:** conducted using CINAHL, Embase, Ovid Medline, PsycINFO, Cochrane Library, University of York Centre for Reviews and Dissemination. Searches were conducted from 1990 until 31^st^ October 2018.
- **Policy and clinical documents:** conducted using [www.evidence.nhs.uk](http://www.evidence.nhs.uk)
- **Grey literature:** conducted using [www.opengrey.eu](http://www.opengrey.eu) and [www.ethos.bl.uk](http://www.ethos.bl.uk) for doctoral dissertations
- **Further strategies:**
  - Using conference proceedings to identify linked full papers or other relevant papers by the authors
  - Discussion with experts regarding potentially relevant papers
  - Hand-searching reference lists of included papers.
