# Supplementary material for: Implementing advance care planning with community-dwelling frail elders requires a system-wide approach: An integrative review applying a behaviour change model
Source: Palliat Med. 2019 May 6;33(7):743–56. doi: 10.1177/0269216319845804 (PMC6620766; doi:10.1177/0269216319845804)
Supplement: Supplementary material [file Supplementary_Data_2_-_28.10.18.docx]

**Supplementary Data 2: Search Strategy**

| **Source** | **Terms** |
| --- | --- |
| **Ovid Medline strategy**  **(tailored for use in PsycINFO, Embase and CINAHL)** | *Advance Care Planning/* OR (advance* care plan* or ACP or advance health-care plan* or advance healthcare plan* or advance health care plan* or advance* medical plan* or plan* ahead or anticipatory plan* or future plan*) OR *Advance directives/* OR (advance* directive* or advance care directive* or Healthcare directive* or Health care directive* or advance* medical directive* or advance statement* or ulysses contract* or advance* decision*) OR *Resuscitation orders*/ OR (ADRT or DNAR or DNR or “do not resuscitate” or do-not-resuscitate or resuscitation order*) OR (end-of-life decision* or end-of-life conversation* or end-of-life discussion* or end-of-life communication or end of life decision* or end of life conversation* or end of life discussion* or end of life communication) OR *Living wills/* OR living will* OR (Preferred place of care or Preferred place of death) OR (healthcare prox* or power of attorney or surrogate decision-maker or surrogate decision maker)  **AND**  *Aged/* OR (Old* person* or old* people or elder* or old* adult*)  **AND**  *Frail elderly/* OR (Frail* or frailty syndrome) OR *Sarcopenia/* OR Sarcopenia |
| **Cochrane Library** | *Advance care planning*/ OR Advance* care planning  **AND**  *Frail Elderly*/ OR Frail* |
| **NHS Evidence, EThOS, open.grey and University of York Centre for Reviews and Dissemination** | (Advance* care planning)  **AND**  Frail* |
