# Supplementary material for: Implementing advance care planning with community-dwelling frail elders requires a system-wide approach: An integrative review applying a behaviour change model
Source: Palliat Med. 2019 May 6;33(7):743–56. doi: 10.1177/0269216319845804 (PMC6620766; doi:10.1177/0269216319845804)
Supplement: Supplementary material [file Supplementary_Data_3_-_18.03.19.docx]

**Supplementary Data 3: Intervention study details**

| **Study design** | **Intervention** | **Main results** |
| --- | --- | --- |
| Baker et al.^61^ (2012) UK  Cohort study of a service improvement. Pre-post outcome measures  **Sample:**  172 home-dwelling frail elders at risk of unplanned hospital admission  IG=96, CG=96 Mean age: IG=80.6 (SD 9.3), CG=79.5 (SD 11.6) | A system-wide intervention including proactive and reactive case management (4.7 WTE extra staff recruited), which rapidly supplied home care (24 hours to six weeks depending on need/local authority capacity), to keep people out of hospital where possible. This included signposting to local trades people for minor works and Citizens Advice Bureau/other voluntary agencies to maximise income. ACP was created from patient discussions, based on Gold Standards Framework and Liverpool Care Pathway, and tailored to personal circumstance e.g. how immediate care was provided (if required), understanding of illness and its trajectory. ACP was reviewed every 6-months (or as requested by person) and copies kept in persons home, GP notes, and with out of hours services at local community hospital. Ambulance service was notified of any DNAR. | **Survivors:** Fewer hospital admissions (p=0.002) Reduced hospital bed days (p=0.020) **Decedents:** Fewer bed days in last 3 months of life (IG: p=0.007; CG: p=0.045) |
| Boettcher et al.^65^ (2015) USA  Service improvement including 6-month pilot and 12-month implementation  **Sample:**  Frail, home-dwelling older people enrolled in specific Medicare plan and actively involved in Case Management. Specific demographics not listed.  Pilot: 38 older people approached. Implementation: 576 approached | Based on Respecting Choices (RC) but adapted for telephone delivery. RC focusses on helping patients to understand their healthcare options, identify gaps in their knowledge, develop questions for their doctor, and create a written plan that represents their goals, values, and healthcare decisions. Intervention used two of the three RC steps:  • First Steps (FS): for healthy adults and those who have never planned. Focusses on the importance of planning; exploring goals of care in the event of losing capacity, and completion of a basic written advance directive. • Last Steps (LS): for adults likely to die in the next 12-months. Goal: to help with specific healthcare decisions, documented as medical orders, to be followed in emergencies. Participants attended standardised, competency-based training. Protocols and scripts were embedded in electronic assessment tools. Participants were provided with a script during the pilot. | **Pilot:** 35/38 indicated interest. 9 FS and 2 LS discussions completed.  **Implementation:  FS:** 198/576 indicated interest, 55 new/updated documents completed.  **LS:** 56/152 discussions completed, 4 LS documents completed.  **Skills assessment**: Case managers' confidence and skills peaked at 3-months, decreasing in most competencies at 6-months (may be due to role changes within the team). |
| Chan & Pang^43^ (2010) China  Quasi-experimental mixed methods feasibility study   Quantitative self-report questionnaires, pre-post outcome measures  **Sample:**  121 cognitively able nursing home residents. IG=59, CG=62. Age: 66-100 (mean 83.5) | Nurse facilitator used a storytelling approach integrating reminiscence therapy and care planning to enable participants to “thread together their past, present and future” as part of the ACP process. Four approximately 1-hour sessions focused separately on: Life stories; Illness narratives; Life views; and EOLC preferences. Participants received a booklet summarising their discussions. Family invited to conference to discuss person’s wishes with the participant and nurse facilitator as mediator. More comprehensive than traditional Advance Directives but the focus of ACP was articulated as future medical decisions. | Increased communication of treatment preference to family/caregivers (p=0.012, OR 4.52).  Positive effect on overall quality of life (p=0.034), and existential distress (p=0.038) Involving families in ACP discussions remained difficult. |
| **Study design** | **Intervention** | **Main results** |
| Chan & Pang^44^ (2011) China  Quasi-experimental mixed methods feasibility study  Qualitative element: semi-structured interviews  **Sample:**  42 cognitively able nursing home residents who had completed the above programme and assessments.  Age: 66–94 (mean 81.33) | As per Chan & Pang (2010) | The qualitative findings established five approaches towards end-of-life care decision-making among frail elders: holding on to life; planning ahead; weighing benefits; avoiding; and deferring |
| Golden et al.^67^ (2009) USA Prospective prevalence study  **Sample:**  530 homebound older adults (clients) without an Advance Directive (articulated as one or more of: living will, durable power-of-attorney for health care, legal guardian, or do-not-resuscitate order) | Case managers attended an in-service on the importance of Advance Directives, the different types of Advance Directives, and study protocol. Clients were contacted to see if they had an Advance Directive. Clients without Advance Directives were asked if they wanted more information.  Clients who did not have an Advance Directive were contacted 3 months later by case managers. If no Advance Directive was in situ, case managers asked clients/caregivers to identify potential barrier(s). | Reminders by case managers were ineffective at increasing Advance Directive prevalence. 8/530 had an Advance Directive (1.6%) at the 3-month follow-up |
| Luptak & Boult^68^ (1994) USA  Service improvement  **Sample:**  34 community-dwelling older people attending a geriatric evaluation & management clinic, receiving medical assistance, and identified as being at high risk of hospital admission  Age range 65-86. | At the first visit a social worker provided verbal information regarding Advance Directives, offered written information about the Minnesota Living Will Act, and answered questions and concerns. Patients were told of three recording options: 1. record preferences and instructions, 2. name proxy, or 3. a combination of both. Patients were given the Minnesota Living Will form to take home and were advised to review and discuss it with family and write down any questions. Patients were also advised relevantly professionals and trained lay-volunteers would be available at subsequent visits to discuss questions and assist if they decided to record an Advance Directive. Advance Directive conversations continued during the next 3-4 clinic visits between the patient and professionals or trained lay-volunteers, including how treatment options fit with personal goals and beliefs. Patient questions about the consequences of specific treatments were answered with a form describing common types of life support and some of their benefits and burdens. Patients who chose to complete an Advance Directive were advised to keep it in an accessible place. Copies were provided for proxies, clinic charts, referring doctors and significant others as requested by patient.  The intervention generally lasted 60-90 minutes (introduction 5-10 mins; patient-professional discussions during three or four subsequent visits over 2-4 months (10-15 minutes per visit); assistance with AD completion where requested (20-35 minutes). | AD completion: 24 patients (70.6%).  Of ADs completed 23 (95.8%) named proxies; 20 (83.3%) specified ≥1 procedures they would want performed on them; 20 (83.3%) specified ≥1 procedures they would not want performed on them. |
| **Study design** | **Intervention** | **Main results** |
| Overbeek et al.^66^ (2018) Netherlands  Cluster randomised controlled trial  **Sample:**  Long-term care residents (90) & community-dwelling adults (111) receiving home care who were frail & able to consent to participate.  IG=101, CG=100. Age range 73-102 (mean 87). | Based on Respecting Choices (RC) but adapted for use in the Dutch context. The intervention focussed on assisting participants to: understand their illness; reflect on goals, values & beliefs; discuss healthcare preferences; & appoint a surrogate decisionmaker. The three intervention core elements were:   - Information provision through leaflets: These including resuscitation, artificial ventilation, artificial feeding & surrogate decision-makers; - Facilitated ACP conversations: Based on scripted interview cards, these included making specific end of life treatment decisions, cards for adults with chronic illness & cards related to euthanasia (legally regulated in the Netherlands), for use if participants raised the topic; - Completion of Advance Directives: Based on Power of Attorney for Healthcare document, this included establishing future medical care preferences & appointing surrogate decision-makers.   The intervention was delivered by eight trained nurse facilitators who had attended a three-day training programme. This included role plays & homework assignments, as well as the legal framework for Dutch Advance Directives. | Completion of Advance Directives and the appointment of surrogate decision-makers increased in the IG. IG=93% completed an Advance Directive and 94% appointed a decision-maker. CG=34% completed an Advance Directive and 67% appointed a decision-maker (p<.001).  No statistically significant differences between IG(–0.26±11.2) & CG(–1.43±10.6) in change scores of the Patient activation measure (p=0.43) or the Quality of Life SF-12.  No differences were found in the in the use of medical care. |
| Patterson et al.^63^ (1997) Canada Randomised controlled trial  **Sample:**  163 chronically ill home-dwelling older people IG=114, CG=49. No demographics given. | Based on 'Let me Decide' (LMD) paperwork. LMD is an institutional and proxy directive that includes four parts: 1. an introduction stating the individual's reasons for completing the directive; 2. a personal healthcare chart; 3. definitions and terms used in the personal healthcare chart; 4. a personal statement where the person describes what they consider an irreversible condition. Four treatment options are offered in the event of life-threatening illness: Supportive/Palliative care; Limited care; Surgical care; and Intensive care. Further sections cover preferences for cardiopulmonary resuscitation and dysphagia management. Different options are chosen based on whether conditions are reversible.  Nurses attended a one-day workshop on LMD. Once they had completed some (number not specified) Advance Directives they attended a refresher workshop which gave the opportunity to discuss issues and concerns. Nurses then used their knowledge of LMD to educate older people on the documentation in a home visit. In subsequent visit the nurse reviewed the Advance Directive of those clients wishing to complete it. 6-months post-trail the nurse visited both IG and CG participants giving IG participants the opportunity to review and make changes to their AD, and CG participants the opportunity to learn about and complete the LMD directive. | 70% of the IG completed an LMD Advance Directive Younger patients (no age specified) were more likely to complete than older (p=0.01).  The study nurse was a significant predictor of Advance Directive completion (p=0.04). Some nurses had all older people complete Advance Directives, some had none. |

| **Study design** | **Intervention** | **Main results** |
| --- | --- | --- |
| Radwany et al.^46^ (2014) USA Randomised pilot study  **Sample:**  Home-dwelling older people >60, who were enrolled in PASSPORT, the long-term Medicare waver programme.  IG=40, CG=40. Age mean: IG=69.5, CG=68.8 | Based on PEACE (Promoting Effective Advance Care for Elders)  Trained care managers contact client’s primary care physician (PCP) and any named specialists to elicit their opinion of appropriate patient goals. PCP are asked to rate patient status from ‘‘many’’ to ‘‘few treatment options still available.’’ Within 3 weeks of enrolment patients receive the first of two standardised in-home palliative care needs assessments. The care manager assesses the client (and family's) biopsychosocial needs and goals. If these are significantly different from those indicated by the PCP, the care manager or other intervention professional, discusses the discrepancy with the PCP before the next visit. This information is used during the 2nd home visit to help inform realistic goal setting. Around 2 weeks after the 2nd visit findings are reviewed at an interdisciplinary meeting with other professionals consulted as required. This team develops an individualised evidence-based care plan based on standardised protocols. A copy is sent to the client’s PCP. The care manager discusses the care plan with the client (and family) to ensure it accurately reflects their goals. Once the client and family agree, the care manager accompanies the client to visit their PCP to review the plan. Once the care plan is agreed by all, the care manager makes another home visit to implement the plan and activate/coach the patient using standardised protocols. This includes completing appropriate legal documents. Clients have access to either the care manager or a hospital-based team member 24 hours a day in case of acute exacerbations. Care manager follows up with the client as needed, at least monthly by phone for 12 months to determine if the goals of care have changed. If the client is re-hospitalised or if there is another trigger the team assessment is updated and goals of care re-evaluated. | Pilot trial so not powered to detect statistical significance. However, at 12 months: Fewer hospital visits (IG: 50% CG: 55% p=0.65) Fewer long-term care admissions (IG: 22.5% CG: 32.5% p=0.32) |
| Schwartz et al.^64^ (2002) USA Randomised pilot study  **Sample:**  61 ambulatory patients either ≥65 years with a chronic or life-threatening disease, or ≥75 years. Participants lived at home or in an independent living facility.  IG=31, CG=30. Age range 65-92 (mean 80) | Based on Respecting Choices. Participants were given two pamphlets that briefly describe ACP and included questions that prompted them to consider what factors affect their personal goals for EOLC and included vignettes regarding situations where ACP may be beneficial. Participants were encouraged to read materials, think about who they might appoint as a proxy, and discuss materials with family members/proxy. Participants (and proxy if appointed) then took part in at least one facilitated discussion with trained nurse facilitator. Discussion focused on enhancing participant understanding of ACP, encouraging them to reflect on their goals for EOLC, communicating wishes with loved ones and developing written plans. Facilitators were trained to administer structured interviews so that all topics were addressed, but also to be able to tailor interviews to participant needs. The intervention generally lasted 1-hour with additional sessions offered as required. Content of the session(s) were recorded on a checklist to ensure all components were included. Sessions included establishing participant's motivation to engage in ACP; eliciting previous healthcare decision-making experiences; level of ACP understanding; perception of health status and likely condition progress; determined participant preferences around personal goals e.g. religious/cultural beliefs and comfort care; explained relevant treatment options; discussed benefits/burdens of relevant life support treatments; supported participant to complete written Statement of Personal Values for EOLC, copies of which were given to the participant, proxy and referring doctor. | Higher congruence between participant and proxy in understanding the participant's EOLC preferences. Complete agreement IG: 76% (19/25) vs CG: 55% (12/22), effect size =0.43. Participants had a greater knowledge of ACP effect size=0.22 IG proxies showed more comfort with potential responsibility, effect size=0.31 |
| **Study design** | **Intervention** | **Main results** |
| Volandes et al.^62^ (2012) USA  Pilot randomised controlled trial  **Sample:**  101 older people ≥65 admitted to a nursing home following hospitalisation  IG=50 CG=51. Mean age IG=79, CG=76 | Based around a previously validated goals of care framework with three levels: Life-prolonging; Limited medical care; Comfort care. Both IG and CG delivered in a quiet room by a trained researcher following a structured script.  **Intervention:** A video decision aid was developed through literature review, the design content and structure reviewed and edited for appropriateness and accuracy by geriatricians, critical care intensivists, palliative care physicians, and decision-making experts using an iterative process. The video was filmed without the use of prompts or stage directions to convey a candid realism in the style known as cinema verite. Participants watched this 6-minute video decision aid on a laptop. The video described the goals of care framework in the same way as the CG but includes visual images of the typical treatments in each level, for example, showing the Life-prolonging option as a ventilated patient in the intensive care unit being treated by respiratory therapists and a simulated cardiopulmonary resuscitation including intubation on mannequin.  **Control:** Participants were read a description of the goals of care framework. Both IG and CG participants were then asked to select which level of care they would prefer if their medical condition worsened while at the nursing home, specifically, ‘‘Imagine that you became very ill and in need of medical treatment, which general approach of medical care would you want provided: life-prolonging care, limited care, or comfort care?’’ Subjects unable to select a level of care were considered uncertain. | Participants in the IG group were more likely to opt for comfort care (unadjusted rate ratio, 1.4; 95% confidence interval, 1.1–1.9, p = 0.02) IG: Comfort n=40 (80%); limited, n=4 (8%); and life-prolonging, n=6 (12%) CG: Comfort n=29 (57%); limited, n=4 (8%); life-prolonging, n=17 (33%); and uncertain, n=1 (2%) |
